# Securing IoT Networks from DDoS Attacks Using a Temporary Dynamic IP Strategy

**DOI:** 10.3390/s24134287

**Published:** 2024-07-01

**Authors:** Ahmad Hani El Fawal, Ali Mansour, Mohammad Ammad Uddin, Abbass Nasser

**Affiliations:** Lab-STICC, UMR 6285-CNRS, ENSTA Bretagne, 29806 Brest, France; ali.mansour@ensta-bretagne.fr (A.M.); mohammad.ammad-uddin@ensta-bretagne.org (M.A.U.); abbass.nasser@ieee.org (A.N.)

**Keywords:** IoT, DDoS, M2M, botnet

## Abstract

The progression of the Internet of Things (IoT) has brought about a complete transformation in the way we interact with the physical world. However, this transformation has brought with it a slew of challenges. The advent of intelligent machines that can not only gather data for analysis and decision-making, but also learn and make independent decisions has been a breakthrough. However, the low-cost requirement of IoT devices requires the use of limited resources in processing and storage, which typically leads to a lack of security measures. Consequently, most IoT devices are susceptible to security breaches, turning them into “Bots” that are used in Distributed Denial of Service (DDoS) attacks. In this paper, we propose a new strategy labeled “Temporary Dynamic IP” (TDIP), which offers effective protection against DDoS attacks. The TDIP solution rotates Internet Protocol (IP) addresses frequently, creating a significant deterrent to potential attackers. By maintaining an “IP lease-time” that is short enough to prevent unauthorized access, TDIP enhances overall system security. Our testing, conducted via OMNET++, demonstrated that TDIP was highly effective in preventing DDoS attacks and, at the same time, improving network efficiency and IoT network protection.

## 1. Introduction

In the forthcoming technological era, characterized by a multitude of devices and machines, Machine-to-Machine (M2M) communication will be increasingly driven by intelligence, minimizing the need for human intervention, especially with the advent of the Internet of Things (IoT). The concept of IoT aims to connect various “things” or “machines”, such as sensors, actuators, and controllers, using different communication technologies, including Long Term Evolution for Machines (LTE-M), Narrow Band-IoT (NB-IoT), Long Range (LoRa), SigFox, etc. This connectivity aims to elevate these machines from being simple and passive to smart, autonomous, and interactive entities. With the inherent capability of machines to sense their environment, this leads to the generation of massive amounts of data. It is predicted that by 2030, the number of IoT devices will reach close to 125 billion [1].

In addition to the significant challenge posed by the sheer number of machines, both academia and industry must also contend with the critical security issue arising from the minimal complexity of IoT hardware, characterized by low processing and storage capabilities. Consequently, these characteristics have attracted malicious attackers to exploit IoT devices and transform them into botnets, enabling the launch of Distributed Denial of Service (DDoS) attacks. DDoS attacks are malicious attempts to disrupt the normal traffic of a targeted server, service, or network by overwhelming the target or its surrounding infrastructure with a flood of traffic. DDoS attacks achieve effectiveness by utilizing multiple compromised machines as sources of attack traffic.

To recall, in September 2016, a spree of massive DDoS attacks temporarily crippled Krebs on Security [2]. The initial attack on Krebs exceeded 600 Gbps in volume, among the largest on record. Remarkably, this overwhelming traffic was sourced from hundreds of thousands of IoT devices under the control of a new botnet named “Mirai” [3]. On 23 February 2021, one of the IP addresses involved in the attack was updated to serve a Mirai variant leveraging, mere hours after vulnerability details were published. Upon successful exploitation, the attackers try to download a malicious shell script, which includes additional infection behaviors such as downloading and executing Mirai variants and brute-forcers [4].

Moreover, using Software Defined Radio (SDR) (ver HackRf One), for example, a potential hacker can now access a wide range of wireless-based communication [5]. However, considering that the generated IoT data can be analyzed and processed in the cloud, we can assume that cyber-attacks (e.g., DDoS attacks) will cause a real security threat for the industry in terms of confidentiality, integrity, and availability.

In comparison to Q4 2021, which had previously held the record for the highest number of DDoS attacks detected by Kaspersky solutions, Q1 2022 witnessed a staggering 46% increase in the total number of DDoS attacks, growing 4.5 times compared to the same quarter in 2021. Additionally, the prevalence of ‘smart’ or advanced and targeted attacks experienced a significant surge of 81% compared to the previous record from Q4 2021. These attacks were not only executed on a large scale but also demonstrated innovation. Examples include a site mimicking the popular 2048 puzzle game to gamify DDoS attacks on Russian websites and a call to build a volunteer IT army to facilitate cyberattacks [6].

In this paper, we propose a new strategy called Temporary Dynamic Internet Protocol (TDIP) that protects IoT networks from DDoS attacks in an efficient way. The proposed strategy works in such a way that all IPs will keep changing periodically to prevent attackers from having enough time to create a botnet.

## 2. Literature Review

IoT enables the collection of data from digital devices, drawing conclusions from these data, and maintaining and improving devices across various domains. However, due to its inherent diversity, IoT is susceptible to numerous security risks, including breaches of confidentiality and integrity, resource shortages, and issues with trust. Consequently, various attacks are launched against IoT systems, with DDoS attacks becoming increasingly common. DDoS attacks, which are challenging to detect, aim to disrupt network servers and resource availability by flooding communication channels from multiple sources using a variety of Internet of Things devices. Consequently, protecting against and analyzing DDoS attacks has become a burgeoning area of research [7].

In this paragraph, we present the state-of-the-art proposed solutions to address the impact of DDoS attacks while spotting the mitigation methods used and the results obtained.

The authors of [8] addressed novel application layer DDoS attacks by analyzing incoming packet characteristics, such as the size of Hypertext Transfer Protocol (HTTP) frame packets and the number of Internet Protocol (IP) addresses sent. They employed a deep learning algorithm called Multi-Layer Perceptron (MLP). Based on simulation results, the proposed algorithm achieved a high detection efficiency of 98.99% for DDoS attacks, effectively safeguarding the IoT infrastructure [8].

In [9], the researchers focused on DDoS attacks in Long-Term Evolution (LTE) networks and presented the Mobility Load Balancing (MLB) algorithm as a solution. This algorithm leverages a mechanism that optimizes resource distribution within the LTE architecture, enhancing traffic flows and network performance without the need for additional backup infrastructure [9].

The authors of [10] concentrated on improving Voice over LTE (VoLTE) and Voice over IP (VoIP) network security. They proposed the Packet Level Restraining Technique (PLRT) approach, which identifies the source of DDoS attacks and prevents the affected node from connecting to the entire network. This method effectively mitigates DDoS attacks and their impacts on the network [10].

In [11], the authors investigated IoT smart home security threats under DDoS attacks. They explored the behavior of IoT devices when targeted by such attacks and examined the reactions of a smart-home personal assistant system. The results demonstrated that integrating a personal assistant into the smart home environment enhances the resistance of user communication with the sensors, providing better protection against DDoS attacks [11].

Additionally, the authors in [12] proposed a lightweight security model for home environments, aiming to protect IoT devices from being enslaved as bots. The authors simulated a scenario and introduced a mechanism to monitor, detect, and filter suspicious traffic patterns. The results showed that the detection mechanism operates smoothly, ensuring network efficiency and avoiding delays [12].

In [13], an explorative defense mechanism was proposed, involving the temporary randomization of IP addresses, Media Access Control Address (MAC) addresses, and port numbers. By implementing this mechanism, attackers are unable to gather network information to launch their attacks. The adaptive randomization of network attributes minimizes overhead, communication errors, and latency while optimizing network operation and maintenance [13].

In [14], the authors introduced an IP Fast Hopping method, which involves real-time changes to the server’s IP address according to a schedule available only to authorized clients. Attackers cannot access this schedule, preventing them from sending requests to the correct IP address. While this method ensures that bots are unable to generate a high enough load to disrupt normal system behavior, we could not find reliable results that prove the concept of this method. Additionally, a security breach could compromise this solution if an attacker gains access to the IP schedule, allowing them to follow the same randomization. Furthermore, this solution is applied only on the server side, whereas our solution covers all devices connected to the network [14].

Based on the previous review, it is evident that DDoS attacks cannot be fully eliminated. Therefore, it is crucial to propose different methods that strike a balance between solution efficiency and applicability in real-world IoT environments.

## 3. IoT Botnet Attacks

IoT botnet attacks are a specific type of cyberattack that specifically targets IoT devices, such as smart home appliances, security cameras, and routers. These attacks involve infecting vulnerable devices with malware, which then allows the attacker to control them and launch coordinated attacks on other devices or networks. The consequences of IoT botnet attacks can be significant and varied, including DDoS attacks that can disrupt websites or services, data breaches resulting in the theft of sensitive information, and, in some cases, even physical damage to the affected devices [15].

A notable example of such an attack is the Mirai botnet. Mirai malware is designed to create botnets by exploiting vulnerabilities in IoT devices, such as default usernames and passwords and a lack of security updates. These devices include cameras, Digital Video Recorders (DVRs), and other smart devices. The Mirai botnet operates by scanning the Internet for IoT devices with default or weak login credentials. Remarkably, the botnet’s source code contains a list of 68 username and password pairs that are commonly used as default configurations by various manufacturers, including DVRs, routers, printers, and IP cameras [16]. These credentials are summarized in Table 1.

Afterwards, Botnets utilize these credentials to launch DDoS attacks against specific websites or networks, overwhelming them with traffic and rendering them inaccessible to legitimate users. One notable aspect of the Mirai botnet is its decentralized architecture, which makes it challenging to dismantle. Furthermore, it possesses the ability to adapt and evolve in response to security measures, posing a persistent threat to IoT security [3]. In addition, Mirai attacks immediately disable commonly used services, such as Hypertext Transfer Protocol (HTTP), upon infecting a device, effectively preventing infected devices from being scanned.

In summary, IoT devices are set to play a crucial role in the coming decades, facilitating human tasks, reducing mission completion times, and lowering operational costs for numerous projects. However, there is a darker side to consider. Gartner predicts that by 2025, cyber attackers will have weaponized operational technology environments to the extent that they can cause harm or even fatalities [17]. Consequently, numerous threats will emerge, potentially jeopardizing the advancement of IoT technology if attackers succeed in infecting various IoT components with malicious code, effectively enslaving these components under the control of hackers or even criminals.

## 4. Temporary Dynamic IP Strategy

The Dynamic Host Configuration Protocol (DHCP) is a widely used protocol that enables the automatic management of IP addresses in a dynamic manner.

In the context of IoT, a DHCP service integrated into an IoT gateway is responsible for assigning IP addresses and related configurations to IoT devices connected to the gateway in Local Area Networks (LANs) and Wireless LANs (WLANs). One crucial parameter in DHCP services is the IP Lease Time (IPLT), which determines the duration for which an IoT device can utilize an assigned IP address. If the DHCP service does not receive a request message from the IoT device within an IPLT, it reclaims the IP address once the IPLT expires [18].

In the literature, rotating IP address solutions have been recently used as a mitigation strategy against DDoS attacks. For example, Inmotion Hosting Company (Virginia Beach, VA, USA) employed this technique as a countermeasure against DDoS attacks [19].

In this paper, we use the concept of dynamic IP while applying a novel strategy known as the Temporary Dynamic IP (TDIP) Strategy. This strategy revolves around periodically changing all assigned IPs to prevent attackers from having sufficient time to infiltrate the network. It achieves this by implementing an IPLT that is shorter than the Penetration Attack Duration (PAD).

**Figure 1:**
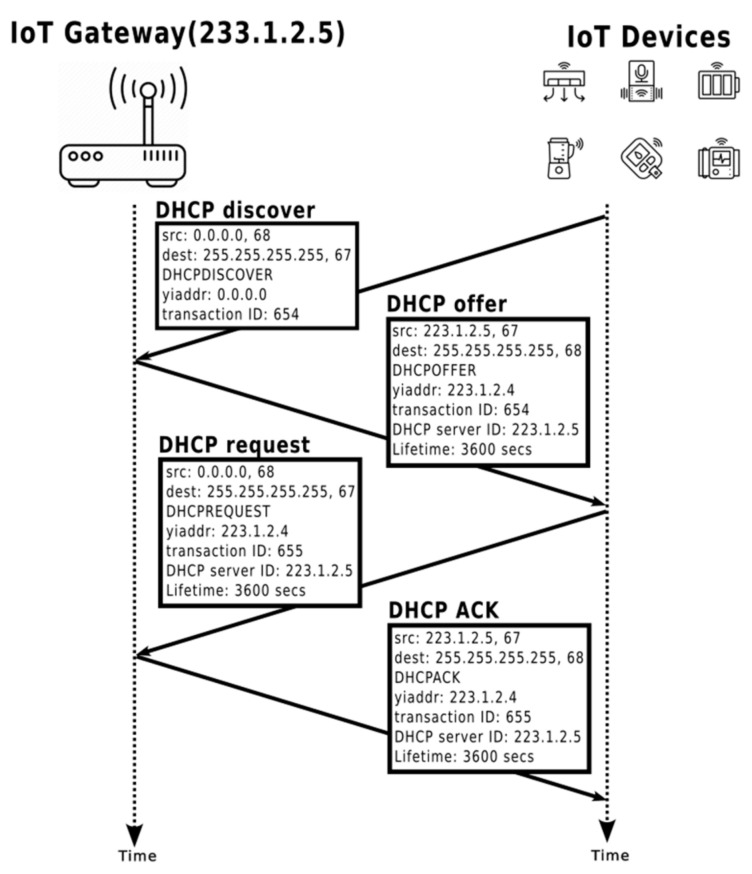
DHCP four steps process: where “src” represents the source IP address, “dest” represents the destination IP address, “yiaddr” is your IP address assigned by the DHCP server to the DHCP client, “Transaction ID” is a random number chosen by the client and used by the server/client to associate messages and responses between a client and a server, and “DHCP server ID” is the IP of the DHCP server.

Similar to standard DHCP service processes, the TDIP strategy follows four steps to assign a temporary dynamic IP to any IoT device, as depicted in Figure 1.

The DHCP process consists of four steps, as outlined in [20]:

1. DHCP Discover: When a newly connected IoT device seeks an IP address from a DHCP service, it broadcasts a DHCP discover message (User Datagram Protocol (UDP) packet) via port 67. This message contains the DHCP discover message, a broadcast destination IP address of 255.255.255.255, and a source IP address of 0.0.0.0. The DHCP client (IoT device) sends this message to the link layer, which broadcasts it to all nodes on the subnet.
2. DHCP Offer: The DHCP service embedded in an IoT gateway responds to the IoT device with a DHCP offer message, again using the IP broadcast address of 255.255.255.255. The offer message includes the transaction ID from the discover message, the proposed IP address for the IoT device, the network mask, and the IP Lease Time (IPLT). The IPLT specifies the duration for which the IP address will be valid, typically ranging from several hours to several days based on network requirements. The network administrator can configure the IPLT manually on the gateway.
3. DHCP Request: The newly connected IoT device sends a DHCP request message to the gateway, confirming the IP address assignment.
4. DHCP Acknowledgment (ACK): The gateway responds to the IoT device with a DHCP ACK message, confirming the assignment of the IP address to the device.

These steps ensure the successful assignment of a temporary dynamic IP address to the IoT device within the DHCP framework.

Once the IoT device has received the DHCP ACK and completed the four-step process, it can utilize the allocated IP address for the duration specified by the IPLT. However, there may be instances where an IoT device may use its IP address beyond the lease expiration. In such cases, DHCP provides a mechanism for the device to renew its lease, allowing it to retain the same IP address for an extended period.

The Temporary Dynamic Internet Protocol (TDIP) strategy is employed to deter attackers from gaining or maintaining access to an IoT network. Under this strategy, if we assume that an attacker requires a certain Penetration Attack Duration (PAD), the TDIP strategy imposes a constraint: IoT devices are granted an IPLT that is shorter than PAD.

By integrating the TDIP strategy into an IoT gateway, all IoT devices undergo frequent changes to their IP addresses, surpassing the rate of the PAD. This perpetual rotation of IP addresses presents attackers with a moving target, significantly complicating their efforts to infiltrate the network. The swift convergence of network IP addresses prevents attackers from establishing a viable botnet to initiate a DDoS attack.

## 5. Comparative Analysis between the TDIP Solution and Previous Solutions

In this paragraph, we revisit the literature and compare the TDIP solution with other solutions proposed in [9,10,12,13,14] in terms of Mitigation Success Rate, CPU/Memory Usage, Network Overhead, and Scope, as shown in Table 2.

- Mobility Load Balancing (MLB) [9] vs. TDIP:

The MLB algorithm aids LTE networks in maintaining resilience during DDoS attacks by reallocating resources from overloaded nodes, ensuring continuous service without requiring additional infrastructure. However, MLB is tailored specifically for LTE networks and may not be suitable for diverse IoT environments. In contrast, the TDIP strategy is specifically designed for IoT networks, employing frequent IP address changes to disrupt attackers. TDIP utilizes the standard DHCP process, facilitating its implementation across various IoT settings.

- Packet Level Restraining Technique (PLRT) [10] vs. TDIP:

The PLRT halts DDoS attacks by identifying and isolating the source of the attack, whereas the TDIP strategy aims to thwart DDoS attacks by constantly changing IP addresses, thus making it more challenging for attackers to exploit IoT devices as bots.

- Lightweight Security Model for IoT [12] vs. TDIP:

The Lightweight IoT model monitors and filters suspicious traffic to protect IoT devices, while the TDIP strategy minimally impacts performance and operates by frequently changing IP addresses to prevent attacks.

- Explorative Defense Mechanism [13] vs. TDIP:

The Explorative Defense Mechanism randomizes network details, such as IP and MAC addresses, to hinder attackers from gathering information. However, the TDIP strategy simplifies defense by frequently changing IP addresses, thereby avoiding potential errors that may arise from randomizing multiple network details.

- IP Fast Hopping [14] vs. TDIP:

IP Fast Hopping involves frequent changes to the server’s IP address based on a secret schedule. In contrast, the TDIP strategy changes IP addresses for all devices, not just the server, making it a more comprehensive solution. Moreover, TDIP mitigates the risk of attackers discovering the IP rotation schedule.

## 6. Simulation, Results, and Result Discussions

We utilize the OMNeT++ open-source simulator [21] in conjunction with a customized INET framework [22], referred to as TDIP_INET, which includes the implementation of the TDIP defense mechanism. This combination is highly beneficial for designing, validating, and exploring new protocols or unconventional scenarios.

In our simulation, we create three scenarios, as shown in Table 3. Each scenario consists of 20 sensors, distributed among three types:

1. Idle Sensors (IS): These sensors solely connect to the DHCP server, simulating devices connected to the network but inactive (sleeping mode).
2. Active Sensors (AS): These sensors communicate with the DHCP server and actively send continuous TCP data. Each sensor sends 400 kbps. Our results are measured based on AS activity.
3. Hacked Sensors (HS): Controlled by the attacker, these bots are present only during the attack scenario, where they disrupt the network using Internet Control Message Protocol (ICMP) flood attacks with an intensity of 4 Mbps.

### 6.1. Simulation Scenarios

The simulation time for all scenarios is set to 1200 s.

#### 6.1.1. Normal Scenario

**Figure 2:**
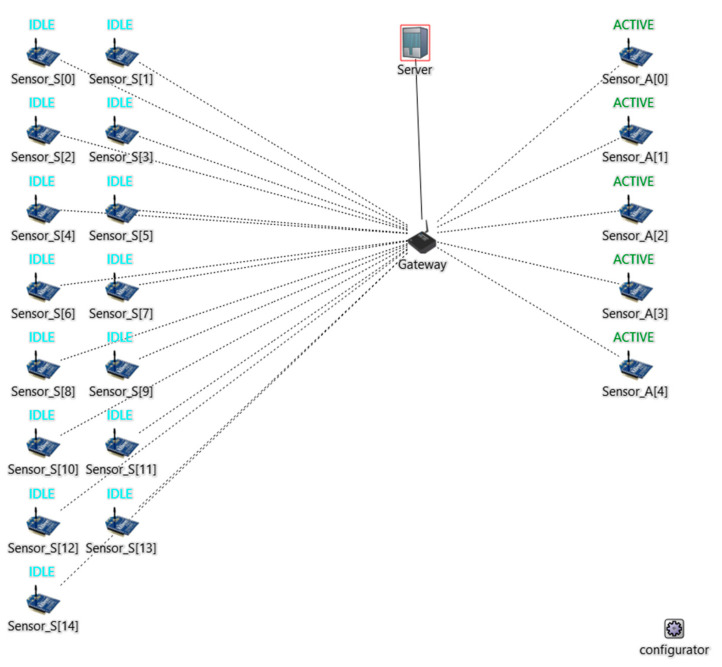
Normal scenario and TDIP solution scenario. The brackets [] here have no relation with the reference citation. They are used here to give numbers to the devices.

This scenario acts as a benchmark. In this scenario, we have 15 IS and 5 AS, as depicted in Figure 2.

#### 6.1.2. Attack Scenario

**Figure 3:**
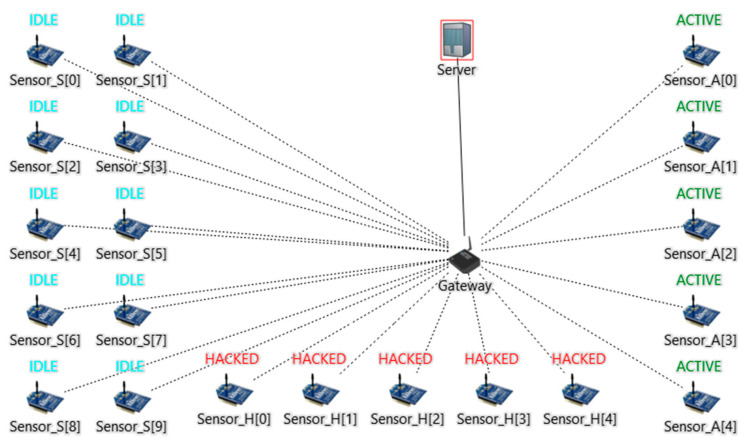
Attack scenario. The brackets [] here have no relation with the reference citation. They are used here to give numbers to the devices.

In this scenario, we assume that an attacker gains control over 5 IS and floods the network with an ICMP flood, as illustrated in Figure 3.

#### 6.1.3. TDIP Solution Scenario

In this scenario, we activate our TDIP defense mechanism, which rotates the IP every 30 ms. We assume that the attacker needed 1 min to gain control over 5 of our IS, turning them into HS. So, we set the lease time for the TDIP server to IPLT = 60 s. In this way, the attacker would not be able to gain control over IS to flood the server.

After outlining the TDIP solution scenario and its implementation, we proceed to analyze the results obtained from our simulations. Specifically, we measure three key metrics: channel utilization, packet loss rate (PLR), and round-trip time (RTT) to evaluate the effectiveness of the TDIP defense mechanism in mitigating network attacks and optimizing network performance.

### 6.2. Results

#### 6.2.1. Channel Utilization

The channel utilization chart is a line chart that shows the percentage of total utilization on the channel over a time interval.

**Figure 4:**
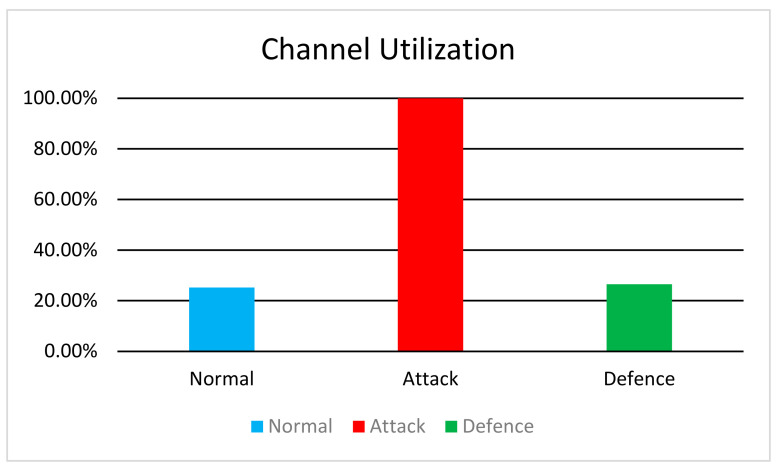
Channel Utilization.

In Figure 4, we observe the following channel utilization rates for a channel with a bandwidth of 10 Mbps.

In the normal scenario, channel utilization is measured at 25.23%, indicating moderate network activity. However, during the attack scenario, the channel utilization peaks at 100%, suggesting severe congestion because of the flooding attack. Notably, during the defense scenario, we observe a decrease in channel utilization to 26.55%, representing a 73.45% reduction from the Attack scenario. This reduction suggests that the TDIP defence mechanism effectively mitigates the impact of the attack, allowing for improved network performance and reduced congestion.

#### 6.2.2. Packet Loss Rate

After examining the simulation metrics, we observe the following outcomes:

Packets Sent: In all scenarios (normal, attack, and defense), the total number of packets sent by AS remains consistent at 569,520 packets. This number is derived from the simulation parameters. Each client is sending data at a rate of 80 Kb per 0.2 s, which translates to 400 Kbps per client. With 5 clients, the total data rate is 2 Mbps. Specifically, we are sending data at this rate for 1200 s, with a Maximum Segment Size (MSS) of 4214 bits.

The calculation is as follows:

Total bits sent=2 Mbps×1200 s=2.4 Gbps

$$Number\;of\;packets=\frac{Total\;bits\;sent}{MSS}=\frac{2.4\;Gbps}{4124\;bits/packet}\approx 569,520\;packets$$

Packets Received: The total number of packets received by the server varies significantly across scenarios:

- Normal: All 569,520 packets sent by AS are successfully received by the server.
- Attack: Out of 569,520 packets sent by AS, only 34,171 are received by the server, resulting in a significant packet loss of 94.15%.
- Defencs: The majority of packets sent by AS (563,767 out of 569,520) are successfully received by the server, indicating minimal packet loss.

Packet Loss Rate (PLR): The PLR is calculated as the percentage of lost packets relative to the total number of packets sent.

**Figure 5:**
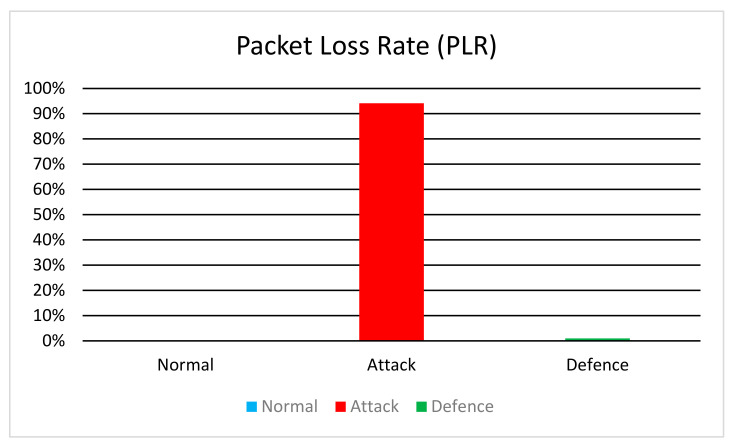
Packet Loss Rate.

In Figure 5, the results highlight the impact of network attacks on packet delivery and the effectiveness of the TDIP defense mechanism in mitigating packet loss.

In the attack scenario, the PLR reaches 94.15%, indicating a high rate of packet loss due to the flooding attack. Conversely, in the Defense scenario, the PLR decreases significantly to 1.01%, demonstrating the successful mitigation of packet loss by the TDIP defense mechanism.

#### 6.2.3. Round-Trip Time (RTT

RTT is the duration in seconds it takes for a network packet to go from a starting point to a destination and back again to the starting point [4].

**Figure 6:**
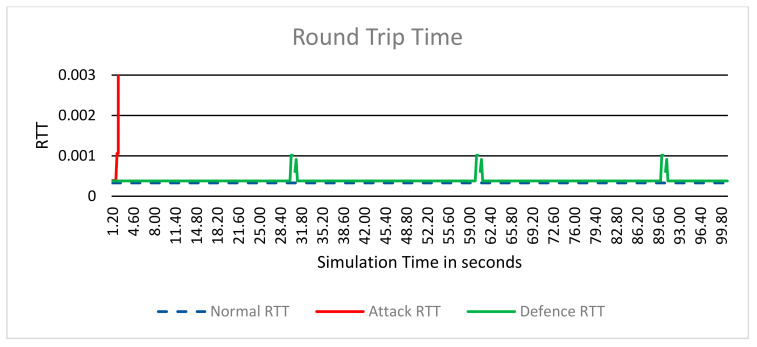
Round Trip Time.

The RTT measurements, depicted in Figure 6, offer insights into the latency experienced by network packets during transmission.

During the normal scenario, the RTT remains relatively constant at 381 μs, indicating consistent and low-latency communication between clients and the server.

In contrast, the attack scenario reveals a stark deviation from the baseline RTT. Initially starting at 381 μs, the RTT abruptly spikes to over 1 s, signifying significant delays in packet arrival. This spike reflects the impact of the network flooding attack on network performance, resulting in increased latency and disrupted communication.

Upon activating the TDIP defense mechanism in the defense scenario, we observe a dynamic pattern in RTT behavior. Initially mirroring the RTT of the normal scenario, the RTT remains stable at 381 μs. However, at intervals of 30 s in simulation time, corresponding to IP address rotations enforced by the defense mechanism, we observe a temporary increase in RTT to around 1 ms, accompanied by a single packet loss. This increase in RTT and packet loss coincides with the IP address change, representing the time required for TCP connection re-establishment. Subsequently, the RTT returns to the baseline value of 381 μs, indicating restored communication stability.

This cyclic pattern of RTT fluctuations aligns with the periodic IP address rotations implemented by the TDIP defense mechanism. The observed stability in RTT between IP address changes underscores the effectiveness of the defense mechanism in mitigating the impact of the attack and maintaining consistent communication performance.

## 7. Conclusions

In this paper, we have introduced the TDIP strategy as a novel approach to mitigate DDoS attacks in IoT environments. The strategy involves periodically changing IP addresses to hinder attackers from gaining sufficient time to create a botnet. By setting the IP lease time to a value lower than the average penetration access time, we can effectively enhance network security during DDoS attacks. Our experimental results demonstrate the effectiveness of the TDIP strategy.

Specifically, our findings reveal a substantial decrease in the packet loss rate during attacks when employing the TDIP strategy. Compared to the total packets sent, the packet loss rate decreased from 94.15% in the attack scenario to 1.01% in the defense scenario. This significant reduction underscores the efficacy of TDIP in preserving packet delivery integrity and ensuring reliable communication amidst malicious activities.

In our future work, we plan to develop a comprehensive framework for implementing a “Redundant IP Pool” strategy. This strategy aims to provide protection for both IoT gateways and IoT devices against DDoS attacks. By incorporating redundant IP addresses and implementing intelligent IP assignment mechanisms, we aim to further strengthen the resilience of IoT networks and enhance their ability to withstand and mitigate DDoS attacks.

## Figures and Tables

**Table 1:** Some well-known passwords are used as default credentials.

Password	Device Type
123456	ACTi IP Camera (ACTi Co., Taipei, Taiwan)
anko	ANKO Products DVR (Anko-Tech Co., Shenzhen, China)
pass	Axis IP Camera (Axis Communications, Lund, Sweden)
888888	Dahua DVR (Dahua Technology, Toronto, ON, Canada)
666666	Dahua DVR
vizxv	Dahua IP Camera (Dahua Technology, Toronto, ON, Canada)
7ujMko0vizxv	Dahua IP Camera
7ujMko0admin	Dahua IP Camera
666666	Dahua IP Camera
dreambox	Dreambox TV Receiver (Dream Multimedia, Lunen, Germany)
juantech	Guangzhou Juan Optical (Guangzhou Juan Optical & Electronical Tech Co., Guangzhou, China)
xc3511	H.264 Chinese DVR (Shenzhen Secumate Technology Co., Shenzhen, China)
OxhlwSG8	HiSilicon IP Camera (Herospeed)
cat1029	HiSilicon IP Camera
hi3518	HiSilicon IP Camera
klv 123	HiSilicon IP Camera

**Table 2:** Comparative Analysis Table between the TDIP solution and previous solutions.

Parameter	MLB [9]	PLRT [10]	Lightweight IoT [12]	Explorative [13]	IP Fast Hopping [14]	TDIP
Mitigation Success Rate	Moderate	High	High	Moderate	High	High
CPU/Mem. Usage	Low	High	Low	Moderate	Moderate	Low
Network Overhead	Low	Moderate	Low	High	Moderate	Low
Scope	LTE networks	Entire network	IoT devices	Entire network	Server-side only	Entire network

**Table 3:** Simulation Parameters.

Parameter	Normal Scenario	Attack Scenario	Defense Scenario
Number of IS	15	10	15
Number of AS	5	5	5
Number of HS	0	5	0
Packet Size for AS	Data: 80 Kb	Data: 80 Kb	Data: 80 Kb
Sending Interval for AS	200 ms	200 ms	200 ms
Defense mode	Deactivated	Deactivated	Activated
DHCP Lease Time	1000 s	1000 s	60 s
Packet Size for HS	No HS	Data: 8 Kb	Eliminated
Sending Interval for HS	No HS	2 ms	Eliminated

## Data Availability

Data are contained within the article.
